# Post Dural Puncture Subdural Hematoma: A Rare Iatrogenic Complication of Neuraxial Anesthesia

**DOI:** 10.7759/cureus.40520

**Published:** 2023-06-16

**Authors:** Nadia Sultan, Muhammad Shah Miran, David Wooldridge, Mudassar Zia

**Affiliations:** 1 Internal Medicine, University of Missouri Kansas City, Kansas City, USA

**Keywords:** severe headache, intracranial hypotension, neuraxial anestehsia, post dural puncture headache, subdural hematomas

## Abstract

Although post-dural puncture headache is a well-known complication of neuraxial anesthesia, subdural hematoma following epidural injection is a rare but potentially under-recognized complication. The following is the case of a young female patient with no significant past medical history who developed a persistent and severe headache following the introduction of epidural anesthesia for labor induction. Imaging demonstrated multiple small subdural hematomas with findings concerning intracranial hypotension. She eventually underwent a blood patch and reported immediate relief from her headache.

## Introduction

A lumber puncture can be used for the administration of regional anesthesia, the intrathecal introduction of certain drugs, and/or the obtaining of cerebrospinal fluid (CSF) for diagnostic purposes. The procedure can be complicated by the development of post-dural puncture headache (PDPH), with a frequency estimated as high as 32% [1], but other complications like post-dural puncture subdural hematomas (PDPsH) are considered rare. During the procedure, CSF leakage may occur as a result of damage to the dura, and sinus veins can be torn, resulting in local bleeding [2]. However, although rare, subdural hematoma following neuraxial anesthesia is a potentially serious complication. Since the symptomatology for PDPH and PDPsH is the same, it is critical for a physician to differentiate them to avoid misdiagnosis and delays in treatment. Herein, we present the case of a young female patient who presented with a severe and persistent headache three days after neuraxial anesthesia. Brain imaging showed subdural hematomas.

## Case presentation

**Figure 1:**
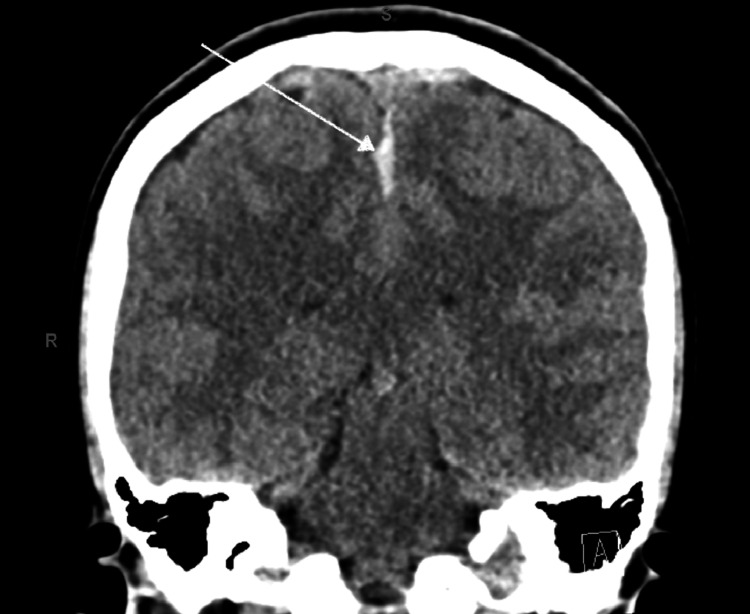
CT head plan coronal view revealing subdural hematoma along the midline falx (white arrow). CT: computed tomography

A 19-year-old gravida one, para one female on postpartum day (PPD) three status post-uncomplicated vaginal delivery with neuraxial anesthesia presented to the emergency department (ED) with a progressively worsening headache since delivery. The headache was bifrontal, constant, 10/10 in severity, and improved with lying down and rest. The headache worsened with any physical activity, and the patient reported sensitivity to bright lights. A chart review from the recent hospitalization revealed that the patient received bupivacain and fentanyl (1.75 mg combined with 15 mcg, respectively) for epidural analgesia, with no apparent anesthetic complications observed during epidural administration. Post-procedure, the block was resolved appropriately, and the patient was able to ambulate independently. In the ED, her pain was unrelieved by acetaminophen (1000 mg PO), ketorolac (30 mg IV), prochlorperazine (10 mg IV), diphenhydramine (50 mg IV), metoclopramide (10mg IV), and caffeine (300 mg PO). The patient was reassessed in three hours again, and in spite of using a multimodal analgesic approach, her headache intensity remained the same. This prompted ED physicians to order further imaging, including CT head, which revealed "high-density material along the falx consistent with subdural blood without significant mass effect" (Figure 1). The patient was admitted to the intensive care unit for frequent neuro checks and further management.

At the time of admission, the patient denied any associated nausea, vomiting, visual changes, fever, recent head trauma, or changes in mentation. She was well-oriented to time, person, and place and had no neurological deficits on physical examination. She was vitally stable, with a body temperature of 97.1 F, a blood pressure of 110/70 mmHg, a pulse of 80 beats per minute, and a respiratory rate of 14 breaths per minute.

**Figure 2:**
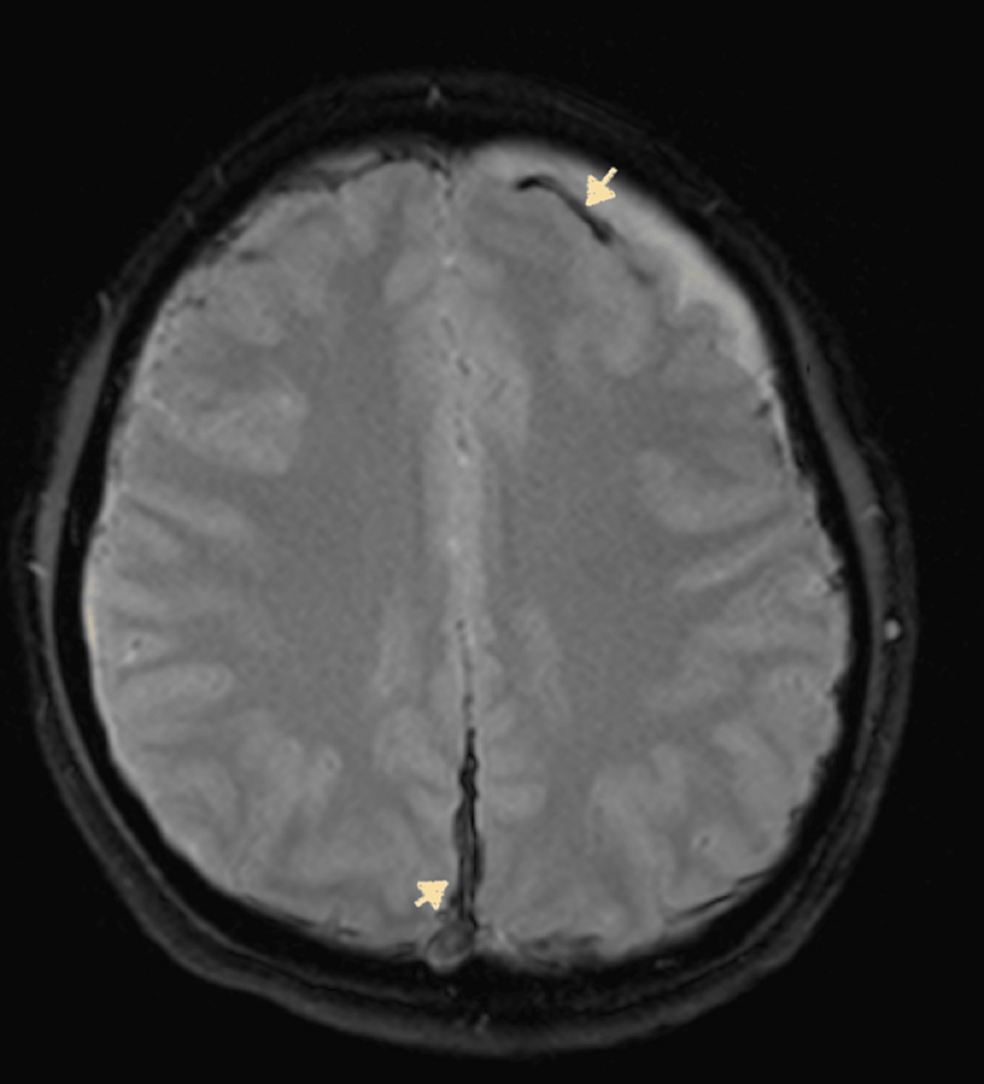
MRI GRE sequence shows subdural hematomas in the frontal cortex and midline falx (white arrows). MRI: magnetic resonance imaging, GRE: gradient recalled echo

**Figure 3:**
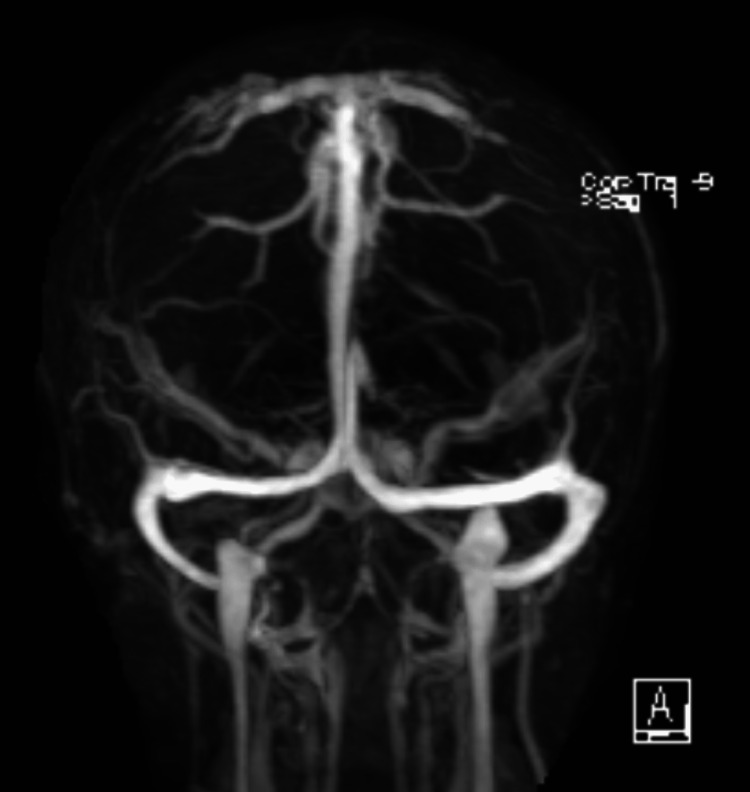
MRV, negative for any aneurysm or arteriovenous malformation. MRV: magnetic resonance venography

**Figure 4:**
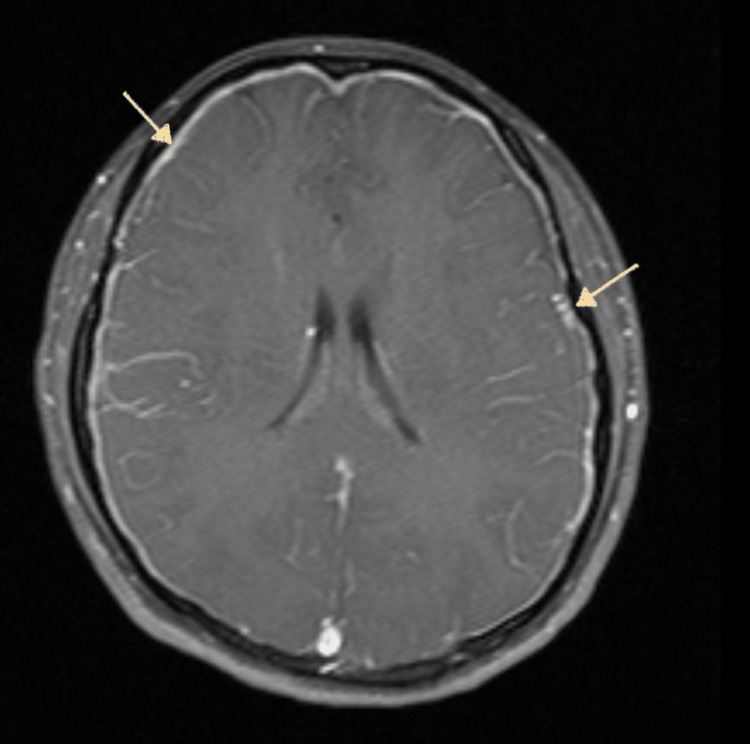
Post contrast T1 sequence shows patchy meningeal enhancement (white arrows) indicating intracranial hypotension.

Neurology and neurosurgery were consulted to help with management. Magnetic resonance imaging (MRI) with and without contrast confirmed the findings of acute bilateral frontal subdural hematomas with additional subdural blood in the midline falx with no mass effect (Figure 2). Subdural hematoma measured up to 5 mm along the midline falx, 5 mm on the left frontal lobe, and 4 mm on the right frontal lobe. Additional imaging with magnetic resonance venography (MRV) was also obtained to rule out dural vein thrombosis but showed engorged cerebral veins at the vertex (Figure 3). Per neurology assessment, subdural hematomas were likely due to CSF leaks secondary to the recent administration of epidural anesthesia, causing intracranial hypotension. The findings of patchy meningeal enhancement on MRI (Figure 4) with reciprocal venous engorgement on MRV advocated the above-mentioned hypothesis as correct. Based on these findings, the neurology and anesthesia teams recommended an epidural blood patch (EBP). The patient was evaluated under anesthesia, and informed consent was obtained about the possibility of inadvertent recurrent dural punctures and further CSF leaks. Almost 20 ml of autologous blood was administered at the L3-L4 epidural space, and the seal was set without any complication.

Post-procedure, the patient reported immediate improvement in her headache (2/10 in the recumbent position and 4/10 on ambulation). The patient was observed for a few more hours in the ICU and was transferred to the floor later. She was monitored for an additional day in the hospital and discharged home in stable condition. After a month, the patient was seen in the obstetrician-gynecologist (OB/GYN) clinic for follow-up. She denied any recurrence of her headaches and had no residual neurological deficits.

## Discussion

Limited case reports of PDPsH have been published; a UK study estimates the incidence at one in 500,000 [3]. Although both complications share similar clinical features, PDPH is far more commonly reported than PDPsH. Similar clinical presentations may delay the diagnosis of PDPsH. A CT head should be considered where clinical suspicion is high or there is a lack of improvement with initial management.

Any procedure that leads to the accidental rupture of the dura can potentially prompt this complication. Continued CSF leakage from the punctured dural site can reduce the effective CSF volume in the subarachnoid space. The reduced intracranial pressure allows the brain to move caudally, stretching the dural veins. In rare cases, these stretched veins get torn, resulting in SDH, like in our case [3,4]. 

As both PDPH and PDPsH result from intracranial hypotension following dural puncture, they share several common risk factors. Low body mass index, the experience of the operator, and the shape and size of the needle are a few of the factors that influence the risk of PPDH and PPDsH [5]. Although conservative measures like strict bed rest, oral or IV analgesia, caffeine, and IV hydration may help relieve symptoms, for both etiologies, EBP has been proven to be the most efficacious treatment modality [3,6]. If symptoms improve with EBP, studies have shown resolution of even thick sub-dural hematomas on their own [7]. However, neurosurgical intervention should be considered in cases of large hematomas or acute changes in mentation, as the chances of uncal herniation are higher in these cases [8]. It has been controversial to perform an epidural blood patch vs. surgical intervention in cases of large hematomas associated with intracranial hypotension. Previous studies have indicated that draining sub-dural hematomas prior to offering EBP can cause a rapid decline in intracranial pressure, further disrupting the bridging veins and leading to the recurrence or worsening of hematomas [7]. However, offering EBP in the first place is a comparatively safer approach, especially in asymptomatic cases. Recent studies support the use of co-syntropin as a viable option in refractory cases [9]. Certain other minimally invasive procedures, like trans-sphenoidal spheno-palatine ganglion block, have been tried by researchers, and the results are promising [10]. However, there is no existing evidence to support the efficacy of these procedures in patients with intracranial hypotension-associated sub-dural hematomas.

## Conclusions

PPDsH is a rare but potentially perilous complication of neuraxial anesthesia. Early recognition and prompt treatment, including an epidural blood patch or timely evacuation of a hematoma, can improve long-term outcomes in high-risk patients.
